# Reciprocal Neighborhood Dynamics in Gun Violence Exposure, Community Health, and Concentrated Disadvantage in One Hundred US Cities

**DOI:** 10.1007/s11524-023-00796-x

**Published:** 2023-10-16

**Authors:** Daniel C. Semenza, Richard Stansfield, Ian A. Silver, Brielle Savage

**Affiliations:** 1 Department of Sociology, Anthropology, and Criminal Justice, Rutgers University, Camden, NJ USA; 2 Department of Urban-Global Public Health, Rutgers University, Newark, NJ USA; 3 New Jersey Gun Violence Research Center, Rutgers University, Newark, NJ USA; 4 Research Triangle Institute, Research Triangle Park, Durham, NC USA; 5 School of Criminal Justice, Rutgers University, Newark, NJ USA

**Keywords:** Gun violence, Community health, Concentrated disadvantage, Longitudinal methods

## Abstract

Gun violence imparts a tremendous human and financial toll on local communities. Researchers have documented extensive mental and physical health consequences of generalized violence exposure but few studies have analyzed the particular impacts of gun violence on community well-being using nationally comprehensive data. We leverage a unique database of almost 16,000 neighborhoods in 100 US cities (2014–2019) to examine how year-over-year rates of gun violence correspond to overall neighborhood well-being and three aspects of community health: (1) health behaviors, (2) physical and mental health status, and (3) health prevention efforts. We simultaneously consider the reciprocal influence of neighborhood well-being on subsequent gun violence while accounting for concentrated disadvantage in communities. The results demonstrate that gun violence is associated with poorer community health in subsequent years, particularly health behaviors and mental/physical health status. Furthermore, we find substantial reciprocal effects for both gun violence and community health in their relationship to neighborhood concentrated disadvantage. These findings highlight the consequential role of gun violence in perpetuating cycles of harm in local communities.

In 2021, nearly 49,000 people in the United States (US) died as a result of a gun injury. This number was driven by a national increase in murders that led to the highest homicide rate in almost thirty years [1]. Research suggests nearly double the number of people killed by a gun each year sustain an injury but survive [2]. Gun injuries are now the leading cause of death for children, having eclipsed car accidents as the most common cause in 2017 [3]. Beyond the enormous human toll, a recent report estimates that gun violence costs Americans more than $550 billion each year including immediate, subsequent, and quality of life costs, comparable to roughly 2.6% of the country’s gross domestic product [4].

Local communities that experience high rates of gun violence have worse collective health [5, 6]. Residents exposed to persistent levels of violence experience allostatic load and greater “wear and tear” on the body, which degrades mental health and exacerbates physical conditions [7]. People that live in high-violence neighborhoods have poorer health behaviors [8], worse mental health [9], higher risk for chronic physical conditions and functional disability [6, 10], and lower rates of healthcare utilization [11]. Local violence exposure generates particular harms among children including poorer cognitive performance [12] and reduced attention and impulse control [13].

Reciprocally, poor community health can contribute to heightened subsequent violence. Alcohol, tobacco, and drug use are associated with greater risk for gang involvement, aggression, and violence [14] while negative health behaviors such as lack of quality sleep are linked to increased risk for criminal behavior that leads to risk for aggression and violence [15]. Beyond poor health behaviors, higher incidence of mental illness in communities increases the risk for violent victimization [16]. More broadly, poor health can degrade social cohesion and support structures that protect against local violence if community members are too unwell to engage in collective activities [17].

Local socioeconomic conditions shape both gun violence and community well-being. Concentrated disadvantage entails a collection of community harms including high levels of poverty, unemployment, family disruption, youth disengagement, and racial segregation [18]. Concentrated disadvantage is one of the strongest community-level predictors of crime and violence, including gun homicides and non-fatal shootings [19]. It is also highly correlated with poorer community health due to a lack of access to healthy food, inadequate public health infrastructure, lack of social capital, and perceived community disorder in especially disadvantaged neighborhoods [20, 21]. Conversely, high rates of violence and poor community health generate further concentrated disadvantage, perpetuating cycles of violence, collective health harm, and socioeconomic disadvantage that endure in highly distressed communities [22].

Despite extant literature, research regarding how intentional, interpersonal gun violence shapes health at the neighborhood level remains limited in three critical ways. First, past studies have focused on neighborhoods in a single location or a handful of cities [6, 8, 10, 23] and there remains no national-level research on how gun violence exposure relates to neighborhood health outcomes. Second, studies are typically limited to a short time frame and rely on cross-sectional analyses [6, 23]. Finally, much of the work to date on gun violence and neighborhood health fails to account for reciprocal (i.e., bidirectional) dynamics in conjunction with the critical neighborhood context of concentrated disadvantage [6, 8–10].

To address these limitations, we analyzed a novel dataset of nearly 16,000 neighborhoods across the largest 100 cities in the US from 2014 through 2019 to examine how combined fatal and non-fatal shootings influence neighborhood health. We simultaneously considered the reciprocal influence of neighborhood health on subsequent gun violence while also accounting for the context of concentrated disadvantage in communities (see Fig. 1 for a conceptual illustration).

**Fig. 1:**
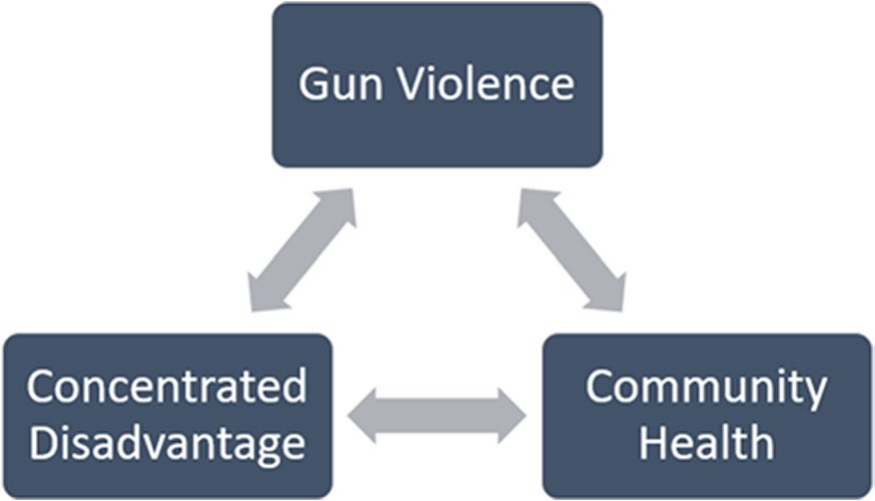
Conceptual reciprocal model

## Methods

We created a neighborhood-level database incorporating 15,845 census tracts embedded in the 100 largest cities in the US from 2014 through 2019 by combining data from numerous data sources. The analytic sample for this study was 14,854 neighborhoods (94% of all neighborhoods) after removing neighborhoods with missing information on health indicators in the CDC data.

### Measures

#### Shootings

The American Violence Project (www.AmericanViolence.org) provides neighborhood-level counts for both fatal and non-fatal shootings for the 100 largest cities in the US based on 2010 census information. We created tract-level counts of total shootings by extracting all incidents and aggregating by census tract and year.

#### Neighborhood Health

We extracted measures from the CDC’s PLACES project, a collaboration between the CDC, Robert Wood Johnson Foundation, and the CDC Foundation. The measures were available annually for the years 2014–2019 at the time of analysis and are routinely used for place-based studies of urban health [24].

We included three indicators of *health behavior* in our analysis: % adults aged 18 or over that sleep less than 7 h a night on average; % adults that currently smoke cigarettes; and % adults who indicated they did not participate in any physical activity or exercise during the past month. Higher percentages for all items are reflective of poorer community health behavior.

We included two indicators of general *health status*: % adult residents who report 14 or more days during the past 30 days during which their mental health was not good; and % adult residents who report 14 or more days during the past 30 days during which their physical health was not good. Higher percentages indicate poorer self-reported health. While self-reported measures capture general perceptions of one’s health, these items are well-established predictors of chronic morbidity, healthcare utilization, and mortality [25].

Finally, we included four indicators of *health prevention efforts* in our study: % adults who report having no current health insurance; % adults who report having their cholesterol checked within the previous 5 years (reverse coded); % men and women over the age of 65 that report having received a core set of clinical preventive services (such as a flu shot in the past year or a colonoscopy in the past 10 years – reverse coded), and % adults who report having been to the dentist in the previous year (reverse coded).

All health measures were subjected to a two-level confirmatory factor analysis (CFA) where the first-order latent factors represented *health behavior* (Factor 1), *health status* (Factor 2)*,* and *health prevention* (Factor 3) while the second-order latent factor represented *overall neighborhood health*. Overall neighborhood health is thus a larger composite of the three constituent factors identified in the CFA model. Within the CLPM (discussed below), overall neighborhood health was created using a CFA, while health behavior, health status, and health prevention were introduced as observed items representing the average percentage across the identified indicators.

#### Concentrated Disadvantage

We created a commonly-used measure of concentrated disadvantage for each census tract that combined the following: % families that live below the poverty line; % residents that identify as Black; % working age civilian population unemployed; and % families headed by a female. Empirically, these measures are shown to be highly correlated while conceptually they capture the accumulation of disadvantage from multiple aspects in a given area [26].

#### Time-Invariant Controls

We measured the distance (in logged miles) to the nearest Level 1 or Level 2 trauma center from the center of each neighborhood [27]. We calculated centroids and distances to the nearest trauma center in QGIS 3.1 We also included % individuals over the age of 18 living alone given the documented relationship between social isolation and community health [28]. Finally, we controlled for the age and gender composition of census tracts via the ratio of female to male population, % males between ages 15 and 24 in the neighborhood, and % in discrete age categories from ages 18 to 84.

### Analytic Strategy

We first produced descriptive statistics and bivariate correlations between the observed items (see Table 1). We then estimated a cross-lagged panel model (CLPM). A CLPM is a longitudinal path model where subsequent observations of two or more constructs (e.g., neighborhood health and gun violence) are regressed on prior observations of the constructs while adjusting for the residual covariation between the constructs at the same time period. By specifying a longitudinal path model in this manner, a CLPM permits the simultaneous estimation of the reciprocal association between the constructs over the analytical time frame while adjusting estimates for the stability within a construct over time. We estimated the CLPM by regressing neighborhood health, gun violence, and concentrated disadvantage at subsequent years on prior observations of the three constructs (see Eq. 1). We replicated this process for every observation of each construct between 2015 and 2019.

**Table 1:** Descriptive statistics and bivariate correlations (2014–2019; *N* = 14,896)

	$\overline{X}$	SD	Bivariate Correlations					
			*Fatal and non-fatal shootings*					
*Health behavior*			2014	2015	2016	2017	2018	2019
2014	27.93	6.87	0.37	0.38	0.36	0.39	0.40	0.41
2015	28.27	6.79	0.37	0.38	0.35	0.39	0.40	0.41
2016	27.37	6.73	0.36	0.37	0.35	0.38	0.39	0.39
2017	28.07	6.69	0.36	0.37	0.35	0.38	0.40	0.40
2018	27.56	6.87	0.36	0.38	0.35	0.39	0.40	0.40
2019	28.15	6.97	0.36	0.38	0.35	0.39	0.40	0.40
*Health status*								
2014	13.10	4.24	0.35	0.35	0.33	0.36	0.37	0.37
2015	13.12	4.01	0.34	0.34	0.32	0.35	0.36	0.36
2016	13.07	3.83	0.33	0.34	0.32	0.35	0.36	0.36
2017	13.53	3.83	0.34	0.34	0.32	0.36	0.37	0.38
2018	13.93	4.04	0.34	0.35	0.33	0.36	0.38	0.38
2019	14.30	4.01	0.35	0.36	0.34	0.38	0.39	0.39
*Health prevention*								
2014	46.76	8.24	0.29	0.29	0.28	0.29	0.30	0.30
2015	46.06	8.27	0.29	0.29	0.28	0.30	0.30	0.31
2016	44.99	8.39	0.29	0.29	0.29	0.30	0.31	0.31
2017	43.81	7.72	0.29	0.30	0.29	0.31	0.31	0.31
2018	43.79	7.26	0.26	0.27	0.26	0.28	0.28	0.28
2019	43.85	7.41	0.26	0.27	0.26	0.28	0.28	0.28
*Fatal and non-fatal shootings*								
2014	0.89	2.02						
2015	1.03	2.28						
2016	1.23	2.95						
2017	1.21	2.67						
2018	1.17	2.52						
2019	1.23	2.59						
*Concentrated disadvantage*								
2014	1.20	0.82	0.41	0.41	0.39	0.42	0.43	0.43
2015	1.16	0.80	0.41	0.42	0.39	0.42	0.44	0.44
2016	1.11	0.78	0.41	0.42	0.39	0.42	0.43	0.44
2017	1.05	0.75	0.41	0.42	0.39	0.42	0.43	0.44
2018	1.01	0.73	0.41	0.42	0.39	0.43	0.44	0.44
2019	0.96	0.71	0.41	0.42	0.39	0.43	0.44	0.44
Miles to nearest trauma center	4.67	8.58	−0.05	−0.05	−0.05	−0.05	−0.05	−0.05
Average percent living alone	50.55	38.42	0.02	0.01	0.00	0.00	0.02	0.03
Average male–female ratio	1.07	0.18	0.13	0.15	0.12	0.14	0.13	0.14
Average percent of males (15–24)	31.16	28.25	0.00	−0.01	−0.01	0.00	0.01	0.00
Percent age 18–34	2.82	1.14	−0.01	−0.02	−0.02	−0.03	−0.02	−0.03
Percent age 35–64	3.77	0.72	−0.10	−0.10	−0.10	−0.10	−0.11	−0.11
Percent age 65–69	0.37	0.20	−0.07	−0.06	−0.06	−0.07	−0.07	−0.08
Percent age 70–74	0.27	0.17	−0.05	−0.04	−0.04	−0.04	−0.05	−0.04
Percent age 75–79	0.20	0.15	−0.03	−0.02	−0.02	−0.03	−0.03	−0.03
Percent age 80–84	0.16	0.14	−0.05	−0.04	−0.05	−0.05	−0.05	−0.05

1 $$\begin{array}{l}{Neighborhood \ Health}_{t+1}={\beta }_{0}+{\beta }_{1}{Neighborhood \ Health}_{t}+{\beta }_{2}{Gun Violence}_{t}+{\beta }_{3}C{oncentrated \ Disadvantage}_{t}+{\beta }_{4}Cov+{\varepsilon }_{it}\\ {Gun \ Violence}_{{t}_{t+1}}={\beta }_{0}+{\beta }_{1}{Neighborhood \ Health}_{t}+{\beta }_{2}{Gun \ Violence}_{t}+{\beta }_{3}C{oncentrated \ Disadvantage}_{t}+{\beta }_{4}Cov+{\varepsilon }_{it}\\ C{oncentrated \ Disadvantage}_{t+1}={\beta }_{0}+{\beta }_{1}{Neighborhood \ Health}_{t}+{\beta }_{2}{Gun Violence}_{t}+{\beta }_{3}C{oncentrated \ Disadvantage}_{t}+{\beta }_{4}Cov+{\varepsilon }_{it}\end{array}$$

We replicated the main CLPM using a correlated traits approach to assess the influence of gun violence exposure on the specific community health outcomes including health behavior, health status, and health prevention. We evaluated model fit by plotting the trajectory of neighborhood health over time, evaluating the variances of the latent factors, and evaluating the R^2^ values [29]. The findings from these evaluations suggest that the CLPM generally fit the data well. We estimated all of the models using the maximum likelihood estimator with robust standard errors using the Lavaan package in R. The raw results for all models presented and accompanying R script are publicly available at https://github.com/ianasilver/Gunviolence_Health.

## Results

**Fig. 2:**
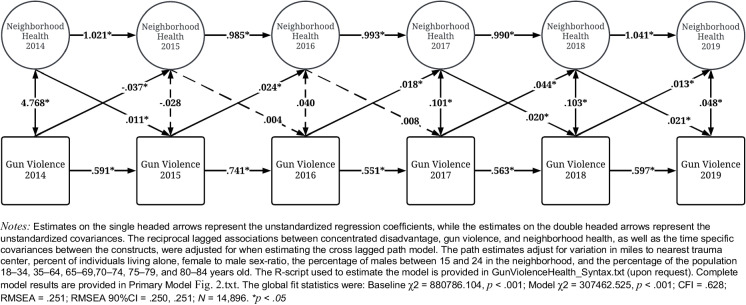
Cross-lagged panel model of neighborhood health on gun violence (2014–2019; *N* = 14,896)

Figure 2 provides the results for the cross-lagged association between general neighborhood health and number of shootings. The level of gun violence in 2015–2018 was positively associated with neighborhood health, where a one-unit increase in shootings was associated with between 0.018 and 0.044 increase in the measure of neighborhood health. Given the reverse coding of all health-related measures, these estimates demonstrate that as gun violence increased within a neighborhood, overall neighborhood health *worsened*. Despite this general pattern of findings, gun violence in 2014 was negatively associated with neighborhood health in 2015. Conversely, worse neighborhood health in 2014, 2017, and 2018 was associated with higher gun violence in 2015, 2018, and 2019.

**Fig. 3:**
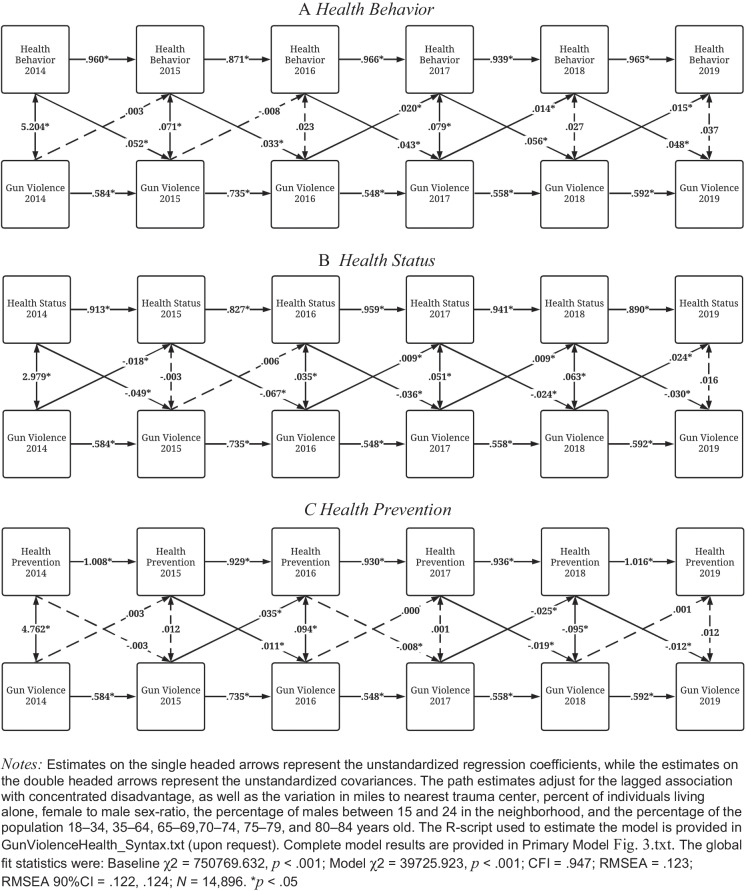
Cross**-**lagged correlated traits panel model of neighborhood health on gun violence (2014–2019; *N* = 14,896)

Figure 3 illustrates the cross-lagged associations between specific health outcomes and shootings using the correlated traits CLPM. In Panel A, increases in gun violence in 2016, 2017, and 2018 were positively associated with health behaviors in 2017, 2018, and 2019. These estimates suggest that as gun violence increased, health behaviors worsened most subsequent years. Conversely, worse health behaviors in 2014–2018 were associated with increases in neighborhood gun violence in 2015–2019.

A similar pattern is found in Panel B, where increases in gun violence in 2016, 2017, and 2018 were positively associated with health status in 2017, 2018, and 2019. Again, these estimates suggest that as gun violence increased, health status worsened during most subsequent years. Notably, gun violence in 2014 was negatively associated with health status in 2015. On the other hand, health status in 2014–2018 was negatively associated with gun violence in 2015–2019. The findings suggest that as health status became better, gun violence increased the subsequent year.

Panel C provides the results corresponding to the reciprocal association between shootings and health prevention. Contrary to Panel A and Panel B, gun violence had a limited association with health prevention efforts and health prevention was not associated with subsequent gun violence.

**Fig. 4:**
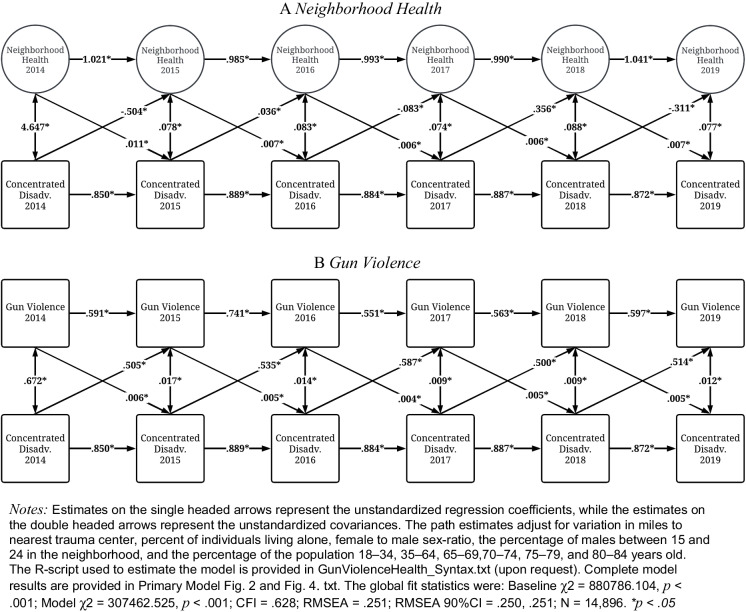
Cross-lagged panel model of neighborhood health and gun violence on concentrated disadvantage (2014–2019; *N* = 14,896)

Finally, Fig. 4 illustrates the lagged reciprocal associations between neighborhood health and concentrated disadvantage (Panel A) and gun violence and concentrated disadvantage (Panel B). These estimates were derived from the initial CLPM and correspond to the results presented in Fig. 2. As shown in Panel A, concentrated disadvantage in 2014, 2016, and 2018 was negatively associated with neighborhood health in 2015, 2017, and 2016, while concentrated disadvantage in 2015 and 2017 was positively associated with neighborhood health in 2016 and 2018. Conversely, worse neighborhood health in 2014–2018 was associated with increases in concentrated disadvantage in 2015–2019.

Finally, as shown in Panel B, increased levels of concentrated disadvantage in 2014–2018 were consistently associated with increased shootings in 2015–2019, while gun violence in 2014–2018 was consistently associated with increased concentrated disadvantage in 2015–2019. These findings suggest that neighborhood health and gun violence influence concentrated disadvantage in a consistent manner, where poorer neighborhood health and increases in gun violence are associated with subsequent rises in concentrated disadvantage.

## Discussion

We analyzed reciprocal relationships between gun violence exposure, community health, and concentrated disadvantage using a dataset of almost 16,000 neighborhoods throughout 100 US cities. Our analyses produced three key findings. First, gun violence was associated with poorer overall neighborhood health in most years from 2014 through 2019. We found a less consistent relationship for the reverse association between neighborhood health and subsequent gun violence. Second, in the correlated traits models (Fig. 3), we found the most consistent relationship between gun violence exposure and both poorer health behaviors and health status. We did not find a consistent relationship between gun violence exposure and health prevention efforts. Finally, both poorer community health and gun violence exposure were regularly associated with higher subsequent concentrated disadvantage. Similarly, concentrated disadvantage dependably predicted subsequent gun violence.

One of the most consistent findings of this study was that gun violence exposure regularly contributes to poorer overall community health. This finding supports the idea that gun violence exposure is harmful to the well-being of whole neighborhoods. Although most people even in high-violence neighborhoods are not involved in gun violence, the dispersive effects of shootings extend far beyond those directly affected. Shootings impact the families, friends, colleagues, and members of community-based organizations working to prevent gun violence. Further, people living in high-violence neighborhoods are likely to hear about or witness shootings in close proximity to their home, even if they do not necessarily know someone personally who has been victimized. Recent research shows that cumulative gun violence exposure, or experiencing multiple forms of indirect and direct gun violence, is particularly linked to poorer mental and physical health [30]. Many of the most violent neighborhoods in US cities have experienced persistently high rates of gun violence for decades, despite a precipitous drop in crime for the country overall throughout much of the past thirty years [17]. In fact, violent crime rates actually rose during this time in many especially disadvantaged communities of color [18]. The neighborhoods that have endured the most violence are also the ones most likely to be comprised of survivors and other residents exposed to shootings over time.

Our correlated traits models (Fig. 3) demonstrate that the specific health harms of gun violence are not limited to mental health outcomes like PTSD, anxiety, and depression that have been the dominant focus of past research [7, 9, 23]. In fact, levels of gun violence most consistently contribute to poorer health behaviors related to sleep, cigarette use, and physical activity. Widespread exposure to gun violence is likely to increase neighborhood fear and distress, making it difficult to practice healthy habits while affecting people exposed in different ways. Residents are also more likely to self-report poorer mental and physical health when living in neighborhoods with higher levels of gun violence exposure during most years in our study. This corroborates the work of past reviews [6, 9] showing that general violence exposure is harmful to both mental and physical health. Our national-level findings across thousands of neighborhoods support the notion that collective gun violence contributes to widespread damages to the self-perceptions of health linked to greater incidence of chronic disease, poorer healthcare utilization, and rate of mortality [25].

Reciprocally, poorer overall neighborhood health was associated with subsequent heightened gun violence in the majority of years during our study. Since the results are less consistent year over year, we are cautious in interpreting these findings although the statistically significant associations all ran in the same direction. Nonetheless, the findings on the influence of community health on gun violence suggest that poorer collective health may create conditions for greater gun violence exposure. It is notable, however, that health status specifically was consistently *negatively* associated with subsequent gun violence in all years. Although this finding was surprising, it may reflect a threshold effect where communities consisting of more residents that report themselves unwell have fewer opportunities for gun violence exposure [31]. Individuals who are mentally or physically ill may not be able to participate in daily activities or freely move around their local neighborhoods, reducing the likelihood of violent encounters or opportunities for victimization.

Surprisingly, we found that concentrated disadvantage did not consistently predict poorer overall neighborhood health year over year. However, when we examined the influence of concentrated disadvantage on the three discrete health outcomes in the correlated traits models (behaviors, status, and preventive efforts), the reason for these mixed findings became clearer (supplemental results available upon request). In these models, concentrated disadvantage consistently and strongly predicted worse health behaviors and health status the following year, which corroborates prior research on health inequities related to poorer socioeconomic conditions [20, 21]. However, the findings were quite mixed for health prevention efforts both in terms of direction and statistical significance. This suggests that concentrated disadvantage may not necessarily be associated with preventive health efforts more common in older populations, but significantly impacts other critical aspects of collective well-being. On the other hand, concentrated disadvantage was a consistent, significant predictor of subsequent gun violence across all years of study, cohering with a large body of research on community conditions and local violence [17–19, 22].

It is striking that both neighborhood health and gun violence exposure consistently correspond with further concentrated disadvantage in all years of our analysis. Although the effect sizes are relatively small from year to year, these effects compound over time and contribute to persistent cycles of harm in local communities. Areas already suffering from concentrated disadvantage may experience greater exposures to gun violence and more harms to collective health. The resultant damages can further entrench local communities in contexts of disadvantage that makes it even more difficult to recover over time. These findings speak clearly to the cyclical nature of socioeconomic disadvantage and collective well-being that have been evident for decades in many of America’s most distressed communities [22]. Further, they implicate gun violence exposure as a key contributor not only to socioeconomic disparities but also inequities in broader community well-being and health.

### Implications for Policy and Practice

Any serious effort to improve community well-being must focus on gun violence reduction and prevention. Beyond direct care for victims of gun violence, community health resources including counseling services should be made available to friends, family members, and local residents in the wake of a homicide or non-fatal shooting [32]. At the same time, many communities lack access or knowledge about resources to assist direct victims of gun violence. Victims Assistance Units embedded within police departments can provide social, financial, and housing services to victims and family members but many community residents are unable to access these resources or do not know they exist. The allocation of resources to support survivor and witness assistance services can mitigate the health harms of gun violence exposure but long-term, equitable investment is paramount to reducing the related health disparities.

It is essential to reduce shootings to improve community health. Researchers have outlined a broad series of evidence-based solutions to reduce gun violence that include gun safety policies, policing initiatives, street outreach programs, improvements to physical environments, and addressing fundamental causes like poverty and residential segregation [33]. Although we cannot summarize all possible solutions here, there is growing consensus that scaling certain policies and programs can effectively reduce shootings and deaths. For instance, evidence suggests that establishing universal background checks alongside permitting license requirements across states would significantly reduce many types of gun violence [34]. Closing background check loop holes can ensure that people who should not have access to a gun cannot get one while reducing the drift of legally acquired guns from federal dealers into secondary black markets [35].

There is substantial evidence that group violence intervention (GVI) programs that support collaborations between policing, community, and social service stakeholders effectively reduce shootings and homicides [36]. GVI and related focused deterrence programs have been successfully implemented in dozens of communities across the US and numerous systematic reviews document their efficacy for reducing crime and violence. On the other hand, community violence intervention (CVI) programs like CureViolence rely on credible messengers to de-escalate conflicts in high-violence neighborhoods and prevent shootings before they happen while working to change local norms around the use of violence. Evidence for the effectiveness of CVI programs remains promising but mixed, in part due to the complexity of maintaining the funding necessary for long-term success and wide variation in program implementation and evaluation methods [37]. However, packaged together with a stronger gun policy infrastructure and well-supported GVI programs, street outreach is a potentially powerful tool for reducing gun violence.

### Limitations and Future Research

There are limitations to our study that provide opportunities for future research. First, our period of study was limited to the years 2014–2019 because these are the years data across all three sources were available. Second, use of the CDC PLACES data did not allow for analyzing change in health outcomes over time across census tracts due to its unique modeling procedure for determining point estimates of local health outcomes each year. To the extent possible, we encourage future researchers to append this dataset to include a longer time period and a greater array of health outcomes that enable measurement of change over time as more data become available.

Third, there are limitations inherent to the use of census tract-level data. For instance, we cannot account for individual selection into particular neighborhoods. Spatial analysis also necessarily relies on a separation of individual units designated by arbitrary boundaries to determine census tracts. As such, the results are sensitive to the definition of units for which the data are collected. Despite this caveat, we rely here on officially collected, governmental data from the US Census Bureau to define the lines of census tracts, which have been used extensively for neighborhood-level analyses.

Finally, there may be variables not available in our data that influence the main relationships examined here. For instance, we could not include rates of other types of crime beyond gun violence because this information is not systematically available at the census tract level. However, guns are now responsible for the vast majority of homicides in the US [2, 3], suggesting that gun violence represents the dominant mode of violence exposure in many communities. Future researchers should also consider including measures of the natural and built environment such as access to parks and green space, abandoned buildings, alcohol outlet density, and density of gun dealers to assess whether they modify the relationships found here.

## Conclusion

The uniquely high rate of gun violence in the US imparts extraordinary human and financial consequences. Our study shows this burden extends beyond the number of lives lost and injuries sustained, degrading the health and well-being of whole neighborhoods. Gun violence and the toll it takes is not equally distributed, disproportionately harming communities that already suffer from high rates of concentrated disadvantage. Although gun violence is likely a critical contributing factor to community health disparities, there are evidence-based policies and programming efforts that can lower shooting rates and effectively reduce these disparities. As gun violence continues to decimate individual lives and whole communities, the failure to act will have enduring consequences for years to come.

## Data Availability

All data are available upon request from the corresponding author.
